# Supplementary material for: Patterns of Intron Gain and Loss in Fungi
Source: PLoS Biol. 2004 Nov 30;2(12):e422. doi: 10.1371/journal.pbio.0020422 (PMC532390; doi:10.1371/journal.pbio.0020422)
Supplement: Table S1 — Also available at http://genes.mit.edu/NielsenEtAl/. (4.3 MB ZIP). [file pbio.0020422.st001.zip › NielsenEtAl/html/118.html]

AN3061.1.NCU06942.1.MG07916.1.FG10964.1


```
 CLUSTAL W (1.82) Multiple Sequence Alignments - Introns Inserted


Sequence 1: AN3061.1	434 aa
Sequence 2: NCU06942.1	441 aa
Sequence 3: FG10964.1	432 aa
Sequence 4: MG07916.1	427 aa
Alignment Length: 444 aa
Number Identitical Residues: 229 aa
Alignment Score (without introns) 10687


MG07916.1 	MPTSWITHSNNGINKITSAVKFDEAGKYQQAFENYMSGCDDLMLAVKKNDAPEETRVALR
NCU06942.1	--MSNTDFLGRAIEQVRKAIEADNAAQYDKAYQLYYQSLELFMLALKWEKNPKS-KEMIR
FG10964.1 	--MS-TNFRDRAIAEVQKAITADHNKEYQKAFDLYMSSMELWVKALKWEKNKAL-KVTMQ
AN3061.1  	--MSNTDFLGRAIDTVKKAIESDNEGEYEKAYQQYYSALELFMLALKWEKNPKS-KEMIR
          	   *   . ...*  : .*:  *.  :*::*:: * .. :  : *:* :.     :  ::

MG07916.1 	RKMAEYLLRAEKLDAHLKAEKSKKEATNGE-SNGAIASN-----~---GQDD--------
NCU06942.1	AKTGEYMDRAEKLKAHLADAEAKRKKPGMVGANGGSTGGTGKGK~EAGEDGNGNGEALDE
FG10964.1 	EKMATYLDRAEKLKQFLANENDPSANGNGKSAMGANGASTSKGK~PAAGEDD--------
AN3061.1  	AKTGEYMDRAEKLKNHLASQDS-RKKPSAVGANGKVSQGSGKGG2KEDDDNE------DA
          	 * . *: *****. .*   .      .  .: *    .:...      :.:      . 

MG07916.1 	DSKKLRNALEGVVLQERPNVKWEDVAGLEGAKESLKEAVLLPIKFPHFFSGKRKPWKGIL
NCU06942.1	DSKKLRSALAGAILQERPNISWDDVAGLEGAKEALKEAVLLPIKFPHLFQGKRQPWKGIL
FG10964.1 	DSKKLRNALSGAILQERPNVRWEDIAGLEGAKETLKEAVVLPIKFPSLFQGKRQAWKGIL
AN3061.1  	DSKKLRSALAGAILSEKPNVKWEDVAGLEGAKEALKEAVILPIKFPHLFTGRRQPWKGIL
          	******.** *.:*.*:**: *:*:********:*****:****** :* *:*:.*****

MG07916.1 	LYGPPGTGKSFLAKAVATEAKSTFFSVSSSDLVSKWMGESER2LVKQLFAMARENKPSII
NCU06942.1	LYGPPGTGKSYLAKAVATEAKSTFFSVSSSDLVSKWMGESER2LVKQLFAMARENKPSII
FG10964.1 	LYGPPGTGKSYLAKAVATEANSTFFSISSSDLVSKWMGESER2LVKLLFSMARENKPSVI
AN3061.1  	LYGPPGTGKSYLAKAVATEANSTFFSVSSSDLVSKWMGESER2LVKQLFNMARENKPAII
          	**********:*********:*****:*************** *** ** *******::*

MG07916.1 	FIDEVDALCGARGEGESEASRRIKTEMLVQMDGVGKDSEGVLVLGATNIPWQLDSAIRRR
NCU06942.1	FIDEIDALCGPRGEGESEASRRIKTELLVQMDGVGKDSKGVLILGATNIPWQLDAAIRRR
FG10964.1 	FIDEIDALCGPRGEGESEASRRIKTEILVQMDGVGNDSKGILVLGATNIPWQLDAAIRRR
AN3061.1  	FIDEVDALCGARGENDSEASRRIKTELLVQMDGVGNDSKGVLILGATNIPWQLDAAIRRR
          	****:*****.***.:**********:********:**:*:*:***********:*****

MG07916.1 	FQRRVHISLPDVAARTTMFKLAVGDTPSSLTNEDYRELAKMAEGYSGSDISNVVNDALMQ
NCU06942.1	FQRRVHITLPDLAARTTMFRLAVGDTHTALKAEDFRELARAAEGYSGSDISIVVQDALMQ
FG10964.1 	FQRRVHIGLPDQNGRARMFKLAIGDTDTALVASDYNVLASKSEGMSGSDIANVVQSALMR
AN3061.1  	FQRRVHISLPDINARMKMFMLAVGSTPCHMTQADYRSLAEQSEGYSGSDISIAVQDALMQ
          	******* ***  .*  ** **:*.*   :   *:. **  :** *****: .*:.***:

MG07916.1 	PVRKMQMATHFKK0IIHEGAEKYTACSPGDPAAVEMTLWQLEGKDLVEPLVTKKDMERAI
NCU06942.1	PVRKIQQATHFKK0VVHEGKQMLTPCSPGDPDAIEMTWEQVPSDELLEPFVDKKDFIKAI
FG10964.1 	PVRKILQATHFKA0VMKDGKRMLTPCSPGDPEKIEMTYDDVSSEELLAPDVQLKDFEMAL
AN3061.1  	PIRKIQTATHYKK0VLHEGQEKLTPCSPGDNGAMEMRWENIEADQLLEPPLVLKDFIKAI
          	*:**:  ***:*  ::::* .  *.*****   :**   :: ..:*: * :  **:  *:

MG07916.1 	NSTRPTVSQDDLKKNAEWTEEFGSEGA
NCU06942.1	KASRPTVSGEDLKRNEEWTKEFGSEGA
FG10964.1 	DDSHPTVSKDDIARQIEWTNEFGSEGA
AN3061.1  	RNSRPTVSQEDLKRNAEWTQEFGSEGA
          	  ::**** :*: :: ***:*******
```
